# The Impact of Verapamil Gel on Efficiency of Tissue Expander Device in Plastic and Reconstructive Surgery

**Published:** 2018-05

**Authors:** Sajjad Hattami, Behzad Khalatbari, Mona Karimi

**Affiliations:** 1 Department of Plastic Surgery, Iran University of Medical Sciences, Tehran, Iran; 2 Burn and Wound Healing Research Center, Department of Plastic Surgery, Shiraz University of Medical Sciences, Shiraz, Iran; 3 Department of Chemistry, Science and Research Branch, Islamic Azad University, Ilam, Iran

**Keywords:** Verapamil, Tissue expander, Plastic surgery, Reconstructive surgery

## Abstract

**BACKGROUND:**

One of the weaknesses of recovering the skin defects by tissue expander device is that it needs relatively long time and it has complications. Since verapamil gel reduces the production of these capsules around silicone prosthesis and reduces the formation of collagen in the capsule areas. The aim of this study was to examine the effect of verapamil gel on efficiency of tissue expander device.

**METHODS:**

Twenty patients were allocated equally into control and case groups based on age, sex, and location of the device. In both groups, the devices were placed in the areas needed and the conditions were identical. During the first operation, the length and width of the flaps and the initial size of prosthesis were determined. In the case group, verapamil gel was used daily, while a hydrogel was used as placebo locally in the control group. Then, other indicators were assessed.

**RESULTS:**

Verapamil gel had no impact on the length, width, area, and size of placed flaps. The opening degree and necrosis of tissue were not improved in patients of case group after using the gel. The effect of verapamil gel in different age groups and gender of people had no significant difference.

**CONCLUSION:**

Verapamil gel was shown not to have any significant impact on efficiency of tissue expander device.

## INTRODUCTION

From fetal life to maturity, growth and expansion of the skin emphasize on this fact that the skin has the ability to adapt to skeletal growth. Growth and expansion of belly skin in a pregnant woman is a clear reason to confirm the physiological sample of tissue expansion. The skin and mucous formed on big benign tumors suggest this fact that we can expand skin and mucosa by non-genetic triggers.^1^ Whether acquired or congenital, benign or malignant, it is better that soft tissue injury to be treated. Sometimes, the injury is so small that it can be easily treated and edge of the wound can be easily stitched and we need to cover the place with free skin grafts or leggy one. Of course, recovered place is in the form is significant.^2^

In 1975, Neumann recommended the use of a subcutaneously implant to recover and reconstruct the deformity of the outer ear. Although this implant was similar common tissue expanders, it was made of rubber and it was filled externally.^3^ Independent work of Radovan and Austad in 1975 by silicone prosthesis expanded the tissue expander idea. Radovan introduced his first experience in the American Plastic Surgeons Community in 1976.^1^ One of the repeated and unpredictable problems in the silicone implants like tissue expanders that contain silicon compounds, is the creation of capsule forms. When these capsules are created around the implants, they cause problems and interferences such as pain and deformity. In some studies, the degree of these shortcomings has been reported approximately 40%.^4^^,^^5^

One of the main reasons for referral of patients to plastic surgeons is to recover the skin deformations that cannot be recovered by medical treatment. Since skin problems are generally visible skin, they cause lack of satisfaction of the people. In addition, in many of the burns in which skin shrink and stretch out, the moving of skin around the face and around the joints that are exposed to stretching gets very painful and limited. Therefore, attempts to find appropriate methods and improving existing methods to recover these defects can help much these.^6^

A relatively new method to recover these deformations is tissue. In 1956, tissue expansion as a reconstructive method surgery of skin was introduced and a balloon was grafted in the lower temporal region of an individual to reconstruct his ear.^7^ Then, the term “tissue expansion” was used for breast reconstruction after breast surgery in 1976.^8^ In Iran, tissue expander device was used for the first time in the plastic surgery part of Imam Khomeini Hospital in 1985. The first tissue expander was undertaken in Imam Khomeini Hospital by donation from America. Currently, tissue expanders are routinely used in several types, depending on the type of deformities.^1^

Nowadays, by placing tissue expander without leaving a patch and ugly scars, soft tissue skin is gradually expanded and its size is increased in a way that the extra of considered place can be easily covered without making nerve, artery, and its physical appearance.^2^ Due to excellent matching of giver area with recipient area, skin expansion provides the best results.^1^^,^^9^ One of the weaknesses of recovering the skin defects tissue expander device is that it needs relatively long time and its complications. One of the most common complications of this procedure is thickening of around the tissue expander device in the form of capsule forms. These capsule forms, containing large amounts of myofibroblasts, are formed due to presence of silicon compounds used in the device that increase the pressure and reduce the efficiency of the device.^4^^,^^10^

Several studies have been conducted regarding treatment of burn wounds of face and neck, using skin grafts, Loco regional flaps, distant flap, and free flaps. It seems that Loco regional flaps are more appropriate, while the main problem is lack of skin in these areas. Due to limited donor tissue adjacent to the burn scars, skin full-thickness grafts and distant flaps were used that do not usually provide desirable results due to marked difference between the grafted and the recipient tissue.^7^ Since no study has been done on humans to investigate the effects of the verapamil drug on the efficiency indicators of tissue expander device, parameters, the aim of this study was to investigate the effect of verapamil gel on efficiency of tissue expander device functionality in the areas of plastic and reconstructive areas in Shiraz, southern Iran.

## MATERIALS AND METHODS

This study is a clinical trial, which is categorized within preliminary human studies considering duration of the study, the cost spent, the number of qualified people who were available, and limitations of study. This study was conducted over a period of one year. The aim of this study was to investigate the impact of verapamil gel on the efficiency of tissue expander device plastic and reconstructive surgery of Shiraz, southern Iran.

The patients were placed equally in case and control groups based on age, sex and location of the device. Within each category, randomly allocation was done based on the location of the device (head and neck, and other parts of the body), age (below thirty years, over thirty years) and gender (male, female). Then, in both groups, devices were placed in the areas needed under similarl conditions in the operating room under anesthesia. The indicators of AB, XY, V, S were measured and recorded in both groups. After recording the above cases and during surgery, qualitative variables of necrosis and the degree of opening were recorded.

In the case group, 0.015 verapamil gel was used, while hydro gel was used as placebo locally in the control group. After one month of surgery, necrosis and the degree of opening of area during the period were recorded and then indictors of AB, XY, V, S were measured again, in order to achieve final result by analyzing recorded data and comparing obtained results in the case and control groups before and after the intervention. This study was conducted on 20 patients who were divided into two equal groups.

All patients who had indication of placing tissue expander device due to trauma, chronic complications of burns or cancer surgery to reconstruct the skin, referred to the available department of plastic surgery clinics were included in the study. In the case of lack of cooperation, lack of consent, unavailability or having indication, patients were excluded from the population of study. In order to investigate the impact of verapamil gel on efficiency of tissue expander device quantitative variables of age, AB, XY, V, S and qualitative variables of necrosis, degree of opening, and gender of patients were studied.

Indicators of AB and XY represent, respectively, the values of length and width of the flap on the tissue expander device on the skin of patient. Indicators of S and V represent, respectively, the values of the area and the size of flap on the tissue expander device on the patient’s skin. Flap necrosis diagnosis was based on the physician’s judgment that in the case of necrosis, a certain percentage was allocated to flap. Indicator (expose) of degree of opening that was related to the skin tear on the device and its opening to be outside.

The mean and standard deviation were used to describe data, and single-sample Kolmogorov test was used to examine the normal distribution of data. To investigate the changes in the length and width of wound, before and after the intervention, paired t-test and Wilcoxon rank mark test were used. To compare the changes before and after the intervention group in the case and control group, covariance analysis was used. To describe variables such as the presence of necrosis and the degree of opening, Chi-Square test was used. To compare indicators of AB, XY, V, S before the intervention in the case and control groups, statistical tests of Mann-Whitney and independent t-test were used. A p value less than 0.05 was considered statistically significant.

## RESULTS

In the case group, none of the patients had skin opening, while one of them had skin opening in the control group. None of the patients had necrosis in the case group, while one patient had necrosis in the control group. These results showed that the verapamil gel had no significant impact on presence of necrosis and degree of skin opening of patients, because no significant difference was found among patients of treatment and control groups. In the analysis performed in the studied groups, no significant difference was observed regarding the age of cases and control subjects. So those who were below thirty years old in both case and control groups were not significantly different, and results were similar for age in groups above 30 years old. 

Analysis was conducted based on gender of subjects showing that there was no significant difference between the subjects of case and control groups, and the degree of recovery was not significantly different in men and women. Tables 1 and 2 shows that the length of flap on tissue expander device before treatment was 6.4±10.8 that after treatment increased to 15.9±21.2, that this increase was statistically significant (*p*=0.005, Z=2.81). In addition, the size of flap on tissue expander device was 11.8±15.3 before treatment that increased to 100.9±106.4 after treatment and this increase was statistically significant (*p*=0.005, Z=2.81). This is true also for the rest of the studied variables.

**Table 1:** Comparison of the mean and standard deviation of indicators of A1B1, A2B2, X1Y1, X2Y2, S1S2, V1V2 in the case and control groups before and after intervention

**Index: Group** **Mean**		**Before the Intervention**		**After the Intervention**		**Z**	**P**
		**SDT**	**Mean**	**SDT**			
AB	Case	10.8	6.4	21.2	15.9	2.81	0.005
	Control	10.9	1.9	17.0	5.2	2.68	0.007
P		---		1		---	
XY	Case	6	3.4	13.4	9.7	2.80	0.005
	Control	6.5	1.8	11.6	4.0	2.68	0.007
P		---		0.971		---	
S	Case	83.9	119.8	418.9	769.9	2.80	0.005
	Control	73.6	33.7	213.9	125.5	2.66	0.008
P		---		0.853		---	
V	Case	15.3	11.8	106.4	100.9	2.81	0.005
	Control	17.8	9.0	74.3	54.0	2.66	0.008
P		---		0.583		---	

**Table 2:** Difference of scores before and after the intervention in the case and control groups

**Index: Group**		**Mean**	**SDT**		
AB	Case	10.4	9.9	0.143	0.215
	Control	6.1	3.6		
XY	Case	7.4	6.6	0.631	0.322
	Control	5.1	2.5		
S	Case	335.0	651.4	1	0.362
	Control	140.3	95.3		
V	Case	91.1	93.4	0.579	0.318
	Control	56.5	51.3		

P* : Due to the high standard deviations, Mann-Whitney U test was used to compare the mean scores difference in the case and the control groups.

P** was calculated using independent t-test.

Due to the high standard deviation of data, non-parametric Wilcoxon rank mark test was used to compare the scores before and after treatment. It should be noted that in this analysis, the results of the t-test and Wilcoxon rank mark test were identical, that is, a significant increase was observed based of both tests. Using covariance analysis, the mean of scores before and after the intervention in the control and case groups was compared. Its results were consistent with results of t-test and non-parametric Wilcoxon rank mark test. For this reason, we did not use the covariance analysis.

**Fig. 1:**
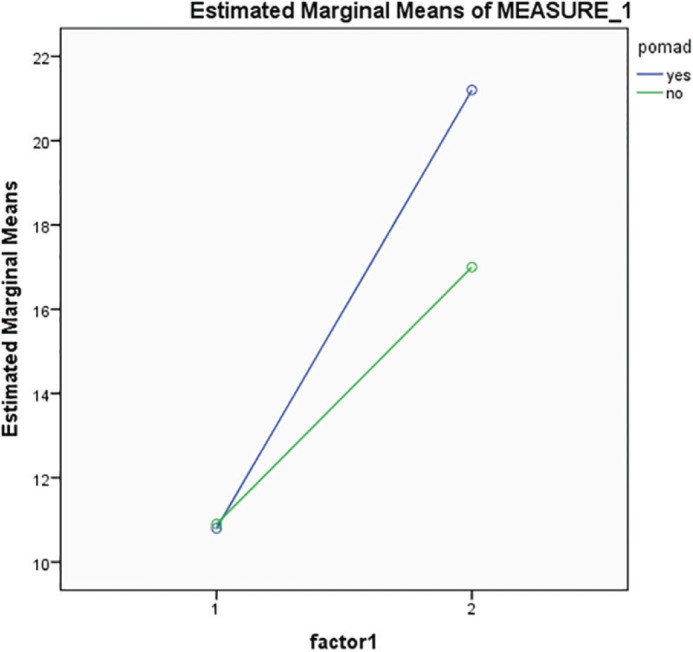
Comparison of mean and standard deviation of indicator AB in case and control groups before and after intervention.

**Fig. 2:**
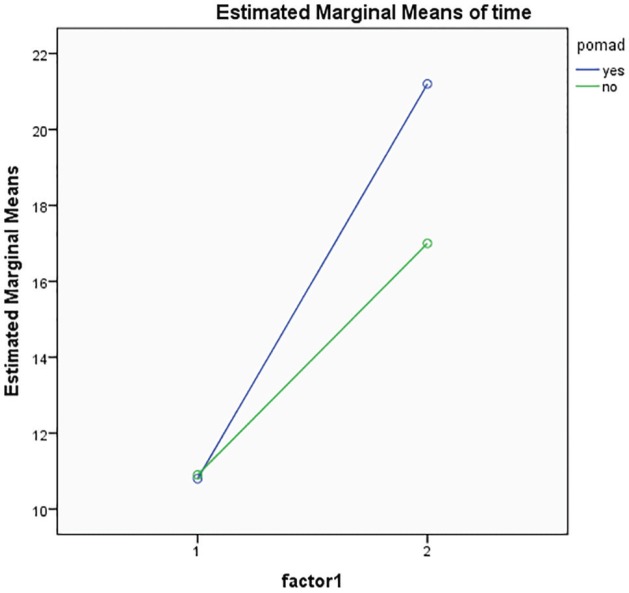
Comparison of mean and standard deviation of indicator XY in case and control groups before and after intervention.

In Figure 1, the blue line represents the case group and green line represents the control group. Figure 1 shows that both groups have similar conditions before the intervention, but for case group after intervention, it is highly increased in comparison to control group. Patients improved after receiving verapamil gel, but this increase was not significant. In Figure 2, the blue line represents the case group and green line represents the control group. Figure 2 shows that both groups have similar conditions before the intervention, but for the case group after intervention, a higher increase is noticed when compared to the control group. Patients improved after receiving verapamil gel, but this increase was not significant.

**Fig. 3:**
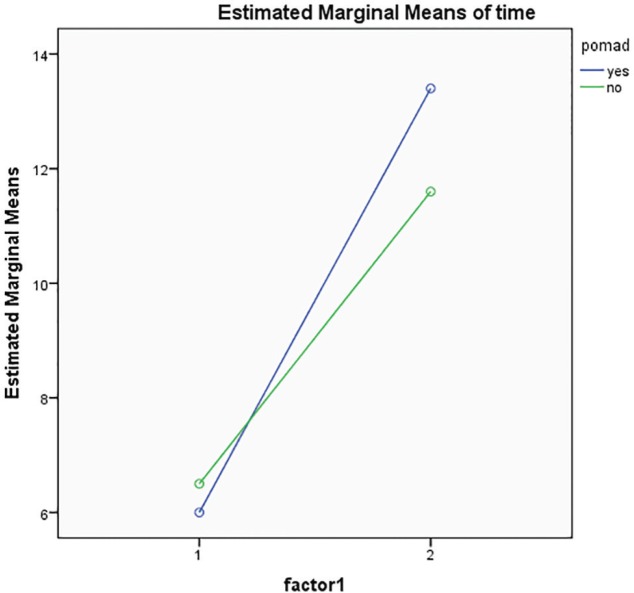
Comparison of the mean and standard deviation of indicators of A1B1, A2B2, X1Y1, X2Y2, S1, S2, V1, V2 in the case and control groups before and after intervention

In Figure 3, the blue line represents the case and the green line the control group. However, we considered the impact of verapamil gel on several indicators (AB, XY, S, V) in the control and case groups before and after intervention. The diagram shows that verapamil gel had no significant impact on improvement of the length, width, area, and size of ulcer of patients, because the mean score of case group increased significantly after intervention.

## DISCUSSION

It was found that verapamil gel had no significant impact on the length, width, area, and size of placed flaps. The degree of opening and necrosis of tissue did not improve in the case group after using the gel. The impact of verapamil gel had no significant difference in different age groups and gender of subjects. Calcium, as an intracellular ingredient, plays an important role in majority of the cells such as fibroblast cells. On the other hand, calcium channel blockers reduce the production of collagen and secretion of fibronectin from fibroblasts.^11^ One of the important effects of calcium channel blockers is an increase in collagen production and activity of growth factor B transformers. Verapamil is one of these blockers and various studies have proven that it has significant impact on the fibroblasts and thus reduces the thickness of the capsules around the silicone implants, including silicone in the tissue expander device.^7^^,^^11^

It was also reported that the verapamil is a calcium channel inhibitor, affecting the reduction of collagen formation in the extracellular matrix.^12^ It was shown that high level of calcium prevents from the adhesion and migration of keratinocytes in vitro, and they claimed that use of this drug is effective in recovering the chronic wounds.^13^ The findings of conducted studies on laboratory animals, such as rabbits, indicate the effect of this drug in reducing capsule forms production around the silicone implants and reducing the formation of collagen in capsule areas, that reduction in production of collagen reduces the production of these capsules.^12^

In some studies conducted on rats, the effect of verapamil on chronic inflammation induced by injection of Freund adjuvant in their paws was examined. Results suggested the effectiveness of verapamil as calcium is known as an important factor in the activation of cells involved in inflammation, calcium channel inhibitors may have anti-inflammatory activity.^14^ It was demonstrated that significant increase in vascular tissues occurs during expansion. They showed that expanded flaps have high degree of survival compared with delayed flaps, that is 17% higher than control flaps in average.^1^^,^^6^^,^^15^

In a study, as the first clinical study, in which the effect of verapamil as a mediator in recovering the wounds was examined, results indicated that verapamil had a statistically significant impact. They stated that verapamil with concentration of 50 μm was an appropriate choice for wounds moderating.^13^ In a study conducted before, the results of massive neck and face burn scars reconstruction using tissue expander were assessed. The authors found that 78% of patients were satisfied with the results of surgery. It seems that use of the tissue expander in the massive neck and face burn scars reconstruction was associated with acceptable results in terms of beauty, and it improved the patient’s appearance.^7^ As verapamil gel impact on the wounds was non-significant, it may be due to the low number of samples and the high standard deviation of the data. Our results showed that patients responded to treatment in highly different ways that may be related to the limitations of this study.

## CONFLICT OF INTEREST

The authors declare no conflict of interest.
